# The Beneficial Effects of Alpha Lipoic Acid Supplementation on Lp-PLA2 Mass and Its Distribution between HDL and apoB-Containing Lipoproteins in Type 2 Diabetic Patients: A Randomized, Double-Blind, Placebo-Controlled Trial

**DOI:** 10.1155/2020/5850865

**Published:** 2020-03-09

**Authors:** Nima Baziar, Ensieh Nasli-Esfahani, Kurosh Djafarian, Mostafa Qorbani, Mehdi Hedayati, Mahshid Abd Mishani, Zeinab Faghfoori, Najva Ahmaripour, Saeed Hosseini

**Affiliations:** ^1^Department of Clinical Nutrition, School of Nutritional Sciences and Dietetics, Tehran University of Medical Sciences, Tehran, Iran; ^2^Diabetes Research Center, Endocrinology and Metabolism Clinical Sciences Institute, Tehran, Iran; ^3^Non-Communicable Diseases Research Center, Alborz University of Medical Sciences, Karaj, Iran; ^4^Chronic Diseases Research Center, Endocrinology and Metabolism Population Sciences Institute, Endocrinology and Metabolism Research Institute, Tehran University of Medical Sciences, Tehran, Iran; ^5^Cellular and Molecular Endocrine Research Center, Research Institute for Endocrine Sciences, Shahid Beheshti University of Medical Sciences, Tehran, Iran; ^6^Department of Clinical Nutrition, School of Nutritional and Food Industry, Shahid Beheshti University of Medical Sciences, Tehran, Iran; ^7^Food Safety Research Center (salt), Semnan University of Medical Sciences, Semnan, Iran

## Abstract

Lipoprotein-associated phospholipase A_2_ (Lp-PLA2) is a new specific vascular inflammation biomarker that is carried by the lipoproteins in the blood and plays a prominent role in the pathogenesis of atherosclerosis. Increased Lp-PLA2 levels and impaired Lp-PLA2 distribution across high-density lipoprotein (HDL) and non-HDL lipoproteins have been reported in diabetic patients, which is associated with the increase in cardiovascular disease (CVD) risk. This study is aimed at investigating the effect of alpha lipoic acid (ALA), as an antioxidant with potential cardioprotective properties, on the Lp-PLA2 mass and its distribution in diabetic patients. In a double-blind, randomized, placebo-controlled clinical trial, seventy diabetic patients were randomly allocated to ALA (1200 mg ALA as two 600 mg capsules/day) and placebo (two maltodextrin capsules/day) groups. The serum levels of total Lp-PLA2 mass, HDL-Lp-PLA2, oxidized low-density lipoproteins (ox-LDL), apolipoprotein A1 (apo A1), lipid profiles, fasting blood sugar (FBS), and insulin were measured, and apolipoprotein B- (apoB-) associated Lp-PLA2 and homeostasis model of assessment index (HOMA-IR) were calculated at the baseline and after 8 weeks of intervention. ALA significantly decreased the ox-LDL, total Lp-PLA2 mass, apoB-associated Lp-PLA2, and percent of apoB-associated Lp-PLA2 and triglyceride and increased the percent of HDL-Lp-PLA2 compared with the placebo group but had no significant effect on HDL-Lp-PLA2 mass, apo A1, lipid profiles, and glycemic indices. There was a positive correlation between the reduction in the ox-LDL level and total Lp-PLA2 mass in the ALA group. In conclusion, ALA may decrease the CVD risk by reducing the ox-LDL and Lp-PLA2 mass and improving the Lp-PLA2 distribution among lipoproteins in type 2 diabetic patients.

## 1. Introduction

Type 2 diabetic (T2D) patients present a high prevalence of cardiovascular events with or without established cardiovascular diseases (CVDs) [1]. The underlying mechanisms in the development of CVD in patients with diabetes mellitus (DM) are complex, and it seems inflammatory processes and oxidative stress play a vital role in the pathogenesis of both of them [2, 3].

Of all the recognized inflammatory markers, lipoprotein-associated phospholipase A_2_ (Lp-PLA2) is a novel specific vascular inflammation and atherosclerosis biomarker that is secreted by macrophages and bound to lipoproteins in the bloodstream [4]. According to Adult Treatment Panel-III (ATP-III) guidelines, measuring the Lp-PLA2 levels is useful for a more accurate diagnosis of coronary heart disease (CHD) risk [5], especially in patients with low-density lipoprotein (LDL) less than 130 mg/dl [6]. A Lp-PLA2 level higher than 200 ng/ml represents that the patient is at a higher risk [5].

Lp-PLA2 may have a role in the pathogenesis of atherosclerosis. This enzyme breakdowns phospholipids in oxidized low-density lipoproteins (ox-LDL) into two proinflammatory and proatherogenic products, lysophosphatidylcholine and oxidized free fatty acids, these inflammatory factors promote atherosclerosis [7–9]. Therefore, it has been proposed that Lp-PLA2 mediates ox-LDL-induced inflammatory responses in the atherosclerotic plaque and is a predictor of coronary events independent of traditional risk factors [4, 8, 10]. However, recently, Lp-PLA2 inhibitor darapladib in two phase III clinical trials in patients with acute coronary syndrome or CHD did not have a protective role in the prevention of further major vascular disease. Indeed, based on several genome-wide association studies (GWAS), the causal relation between Lp-PLA2 and CHD remains controversial [11–13].

Some studies have found that Lp-PLA2 and ox-LDL levels in diabetic patients are higher than those in healthy individuals [14]. Higher levels of this enzyme are associated with the increased risk of incident of CHD among diabetic patients, so Lp-PLA2 could be considered a potential therapeutic target to decrease atherosclerotic risk and the development of cardiometabolic complications [7, 15, 16]. Given the proposed role of ox-LDL in the production of Lp-PLA2, it has been suggested that reducing Lp-PLA2 mass by ox-LDL-lowering intervention may decrease the risk of CVD in diabetic patients [17–19].

On the other hand, it has been proposed that Lp-PLA2 may have both proatherogenic and antiatherogenic features depending on the type of lipoprotein which this enzyme is bound. About 70% to 80% of the total serum Lp-PLA2 bound to LDL, whereas the 20% to 30% is associated with high-density lipoproteins (HDL) [15, 20]. It is assumed that HDL-associated Lp-PLA2 has an anti-inflammatory and vasculoprotective activity, whereas Lp-PLA2 bound to apoB lipoproteins is proinflammatory and atherogenic. Therefore, the relative distribution of Lp-PLA2 could be useful in the assessment of CVD risk [21, 22]. An altered distribution of Lp-PLA2, with a proportionately less mass of HDL, has been reported in diabetic patients [23].

Alpha lipoic acid (ALA), also known as thioctic acid, as an essential cofactor of dehydrogenase enzymes, is involved in mitochondrial macronutrient metabolism. ALA has antioxidant characteristics that unlike most other antioxidants, is both water- and fat-soluble; thus, it can reach easily into tissues composed of fat, such as the nervous system as well as those mainly of water such as the cardiovascular system, so considered a “universal antioxidant” [24–26]. ALA exhibits direct free radical scavenging properties and with respect to lower redox potential (-0.32 v) compared to many antioxidants, such as carotenoids, vitamin E, vitamin C, and glutathione, and has more antioxidant potency and so, it is able to regenerate these antioxidants [24, 27–29]. ALA has beneficial effects in preventing or relieving symptoms of diseases related to oxidative stress such as diabetes and cardiovascular disease [25, 30–32]. Animal and in vitro studies have suggested antiatherogenic properties for ALA; however, few clinical studies have been conducted in this regard [33, 34]. ALA may protect against atherosclerotic cardiovascular diseases (ASCVD) by inactivating reactive oxygen species (ROS) and chelating metal ion, subsequently preventing LDL oxidation and improving lipoprotein metabolism [35–37].

In in vitro studies, induction of oxidative stress via ox-LDL and ROS has been associated with increased Lp-PLA2 gene expression in human monocytic cells (THP-1). On the other hand, it has been shown that ALA suppressed ROS-stimulated Lp-PLA2 gene expression in this immune cell [19, 38]. Thus, we hypothesized that ALA can reduce the Lp-PLA2 mass and its distribution among HDL and apoB-containing lipoproteins in diabetic patients.

To the best of our knowledge, no intervention study has examined the effect of ALA on Lp-PLA2 mass and its distribution among lipoproteins in type 2 diabetes mellitus patients. Therefore, this interventional study is aimed at investigating the effects of ALA supplementation on Lp-PLA2 mass and its distribution between HDL and apoB-containing lipoproteins and ox-LDL in type 2 diabetes mellitus.

## 2. Materials and Methods

### 2.1. Participants

This study was conducted from May to August 2019 at the Diabetes and Metabolic Disease Clinic of Endocrinology and Metabolism Research Institute, Tehran University of Medical Sciences. T2D patients who were willing to participate in the study were invited based on their medical records for the last three months. After assessing for eligibility, 70 non-insulin-dependent diabetes mellitus (NIDDM) patients with a body mass index (BMI) between 18.5 and 29.9, aged between 40 and 60 years old, diagnosis of Type 2 DM for at least two years, and HbA1C < 7% were enrolled in the study.

The following were criteria for the exclusion of subjects from the study: (a) history of heart attack, angina pectoris, stroke, and other chronic diseases and contagious diseases in the past year; (b) use of smoke and alcohol during the last three months prior the study; (c) changing in medications received in the previous three months; (d) using any drug other than metformin, sulfonylurea, statins, angiotensin-converting enzyme inhibitors (ACEI), angiotensin receptor blockers (ARB), and aspirin at doses of 80 mg or less; (e) taking any supplements containing antioxidants and omega-3 in the past three months (at least once a week); and (f) pregnancy and breastfeeding. Also, the exclusion criteria during the study were the unwillingness to continue the cooperation, change in any of the entry criteria, and possible side effects of taking supplements and nonregular consumption of supplements (less than 80% of the supplements).

The aim and procedure of the study were described to each subject, and the voluntary written informed consent was signed before the initiation of the trial. This clinical trial was registered with the Iranian Registry of Clinical Trials (https://www.irct.ir/trial/30483, identifier: IRCT20180407039219N1. Registered on 27 April 2018) and was approved by the ethical committee of Tehran University of Medical Sciences, Tehran, Iran (reference number IR.TUMS.VCR.REC.1396.4583).

The sample size was calculated based on a significant mean difference of Lp-PLA2, which as observed in previous studies [39], with a 95% confidence level and 80% power, a total of 32 subjects were estimated for each group. Assuming the 10% dropout rates, the sample size was increased in 35 subjects in each group.

### 2.2. Study Design and Intervention

This study is a double-blind, randomized, parallel, placebo-controlled clinical trial. Seventy diabetic patients were randomly allocated in a ratio of 1 : 1 into the intervention and control groups by permuted block randomization. We used stratified randomization to match participants based on sex and menopausal in both groups.

Subjects in the intervention group (*n* = 35) and the control group (*n* = 35) received 1200 mg of ALA supplement per day (as two 600 mg capsules) and placebo capsules (maltodextrin), respectively, for eight weeks. In order to better absorption and minimize possible gastrointestinal complications, all patients were asked to take one capsule, two times daily 30 minutes before meals. The shape, odor, taste, and packaging of the supplements and placebo capsules were similar. For blinding the researcher and patients, the capsules' containers were marked with A and B by an expert in the clinic who was blind to the study. ALA supplements (Support Nutrition Company, USA) are supplied by Darou Darman Sepehr Co.

For improving the patients' adherence to the study, we called them up and reminded taking capsules; also, we asked about possible side effects such as skin sensation, fever, and gastrointestinal discomfort. Due to difficulty in measuring the ALA concentration, for evaluating the patients' compliance, the remaining capsules were counted at the 4^th^ and 8^th^ week. We asked the participants to continue their routine lifestyle, including dietary and physical activity habits during the study and notify us in case of changing the lifestyle or drugs for any reason.

We measured primary outcomes including the Lp-PLA2 mass, the percent of HDL-Lp-PLA2, and the percent of apoB-associated Lp-PLA2 and secondary outcomes including the fasting serum levels of ox-LDL, insulin, glucose, triglyceride (TG), total cholesterol (TC), LDL, and HDL and homeostasis model of assessment index (HOMA-IR) at the baseline and after 8 weeks of intervention.

### 2.3. Blood Sampling and Laboratory Measurements

Venous blood samples (10 ml) were drawn by venipuncture after 12-hour fasting at baseline and the end of the study. The blood samples were centrifuged for 10 minutes at 3000 rpm, and serum aliquoted into separate microtubes and were stored in -80°C until biochemical analyses.

Serum levels of glucose and total cholesterol, LDL, HDL, and triglyceride were measured by an autoanalyzer instrument (BT 1500, Biotecnica Instruments, Italy), using commercial kits (Pars Azmoon, Co., Iran). The intra- and interassay coefficients of variations (CV) for glucose, TC, HDL, LDL, and TG were 4.7% and 5.5%, 1.6% and 1.1%, 0.8% and 1.8%, 0.6% and 1.2%, and 1.53% and 1.6%, respectively. Serum insulin was measured by using an enzyme-linked immunosorbent assay (ELISA) kit (Monobind, Uppsala, US). The intra- and interassay CVs for insulin were 5.6 and 4.9%, respectively. Insulin resistance was determined by calculating the HOMA-IR using the following formula: fasting glucose concentration (mg/dl) × fasting insulin level (mU/l)/405 [40]. The serum level of ox-LDL was measured by using an ELISA kit (Shanghai Crystal Day Biotech Co., Ltd., China). CVs in intra- and interassay were 8 and 10%, respectively. Lp-PLA2 mass was measured by using an ELISA kit (diaDexus, Inc. South San Francisco, CA, USA). The intra- and interassay CVs were 5.5% and 7.5%, respectively.

### 2.4. Lp-PLA2 Distribution Determination

To determine the Lp-PLA2 distribution across HDL and apoB-containing lipoproteins, after precipitating the apoB lipoproteins from serum, HDL-Lp-PLA2 mass was measured using a Lp-PLA2 enzyme-linked immunosorbent assay (ELISA) kit (Changhai, Cristal Day Biotech Co., Ltd., China) on the obtained supernatant. The reagent containing 0.55 mM phosphotungstic acid and 25 mM MgCl2 (Sigma-Aldrich, ST. Louis, MO) was used to precipitate the apoB lipoproteins. 200 microliters of serum sample was mixed with 500 microliters of precipitation reagent and incubated for five minutes at room temperature and centrifuged at 1500 g for ten minutes [41]. 200 microliters of apoB-depleted supernatant that contains only HDL were carefully separated without disrupting the precipitate and assayed for HDL-Lp-PLA2 mass.

Finally, apoB-Lp-PLA2 calculated by total Lp-PLA2 mass minus HDL-Lp-PLA2 [42]. The relative distribution of Lp-PLA2 was expressed as the percent of HDL-Lp-PLA2 [(HDL-Lp-PLA2/total Lp-PLA2 mass)^∗^100] and the percent of apoB-associated Lp-PLA2 [(apoB-associated Lp-PLA2/total Lp-PLA2 mass)^∗^100].

### 2.5. Anthropometric Assessment

Body weight was measured by a calibrated scale with 0.1 kg accuracy (Seca, Hamburg, Germany) with barefoot and minimal clothes. Height was measured by a wall-mounted tape, barefoot and at a straight standing posture with 0.5-centimeter accuracy. BMI was calculated by dividing weight in kilograms by height in meters squared.

### 2.6. Assessment of Dietary Intake

To assess participant's food intake, we collected a 24 h food recall at the baseline and end of the study. The nutrition professional asked the patients to recall their intake by using a specific set of questions to gain as much detailed information as possible. To take the best result, for better estimating the portion size of the food and drinks, we used the Household Measures and Food Model booklet. Dietary intake was analyzed in terms of energy and macro- and micronutrients (with a focus on micronutrients with antioxidant activity) intake by Nutritionist version 4 that was modified for Iranian foods.

### 2.7. Assessment of Physical Activity Levels

To evaluate the physical activity levels, the Iranian version of the short form of International Physical Activity Questionnaires (IPAQ) was used at the baseline and the end of the study [43, 44]. The short form of IPAQ includes seven questions that explore walking and moderate-intensity and vigorous-intensity activity during the past seven days. Frequency (measured in days per week) and duration (time per day) are collected separately for each specific type of activity. To analyze the activities, MET-minute (metabolic equivalents per minute) score were used. The MET-minute score was computed by multiplying the MET score by the minutes performed and used categorical score.

The patients with at least 3000 met/minute score obtained from the combination of all the activities were considered category 3 (high) and with at least 600 met/minute score were considered category 2 (moderate), and patients not included in category 2 or 3 were considered category 1 (low) [45].

### 2.8. Statistical Analysis

The obtained data were analyzed with SPSS, version 21 (SPSS Inc. Chicago, IL, USA). An Intention-To-Treat (ITT) approach was used for data analysis. In this approach, all the enrolled participants who take ≥one capsule were included. The multiple imputation method was used for predicting the missing values at the end of the study. In this method, we used a linear regression model to predict variables at the end of the study, by using values of the baseline and study group as the predictor. Results were expressed as mean ± standard deviation (SD) and percentage for quantitative and qualitative variables, respectively. The Kolmogorov-Smirnov test detected the normality of variables, and if the distribution of a variable violated from the normal distribution, an appropriate transformation was performed. At the baseline, quantitative variables were analyzed by independent sample *t*-test and qualitative variables by chi-square test. A paired *t*-test was used to compare the mean variables before and after the intervention in each group, and two-way repeated measure ANOVA was used to identify the effect of the intervention on variables. To examine the correlation among variables and their changes, a Pearson correlation analysis was used. A *P* value of less than 0.05 was considered statistically significant.

## 3. Result

**Figure 1:**
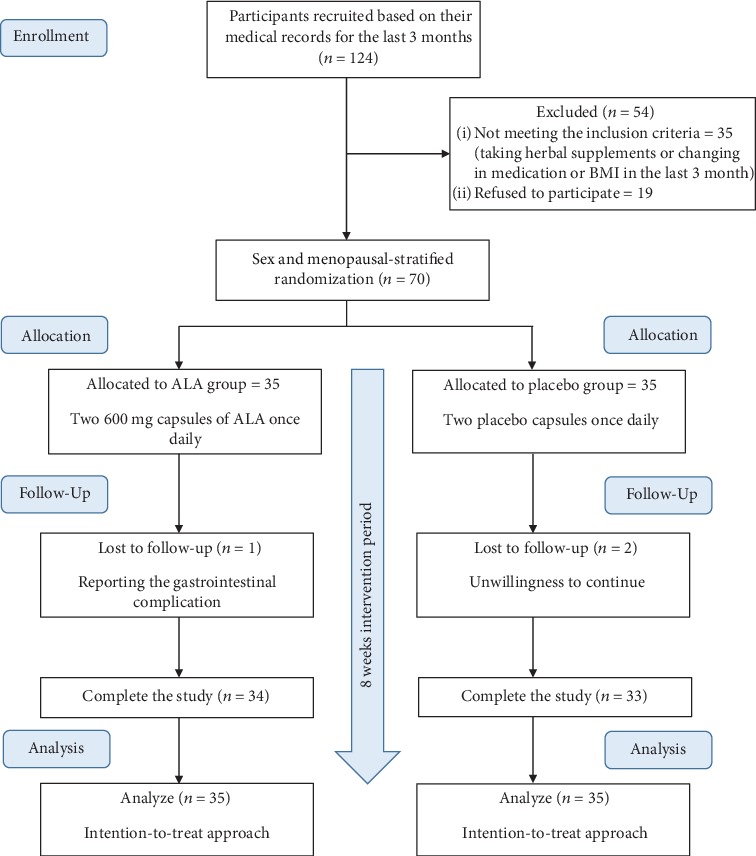
Study flow chart.

Seventy diabetic patients were enrolled in the study and randomly allocated to the ALA group (*n* = 35) and the placebo group (*n* = 35). Three patients dropped out from the study, one from the ALA group (because of reporting the gastrointestinal complication) and two from the placebo group (because of unwillingness to continue). The study flow chart is depicted in Figure 1. After counting the returned capsules among those who completed the study, it was revealed the patients' compliance was high; about 98% of capsules in the ALA group and 96 percent in the placebo group were consumed. Since we applied the ITT approach, all the 70 participants were entered into the statistical analysis.

### 3.1. Baseline Characteristics and Dietary Intake

As depicted in Table 1, at the baseline, there were no substantial differences between the ALA and the placebo group in terms of patients' main characteristics. As expected, no significant differences were found in the BMI and physical activity level at the baseline and end of the study within and between the study groups. Dietary intakes are presented in Table 2. Energy and macro- and micronutrients intake were statistically similar between and within the two groups at the baseline and the end of the study.

### 3.2. Effect of ALA Supplementation on Lipid Profiles and Glycemic Indices

Lipid profiles (TC, LDL, HDL, and TG) and glycemic indices (glucose, insulin, and HOMA-IR) of participants at the baseline and after eight weeks of intervention are depicted in Table 3. There were no significant differences in terms of HOMA-IR, fasting serum glucose, insulin, TC, LDL, and HDL before and after the intervention in both groups. At the baseline of the study, no significant difference in serum TG was observed between the two groups. At the end of the study, TG reduced significantly in the ALA group (*P* < 0.001) and the significant time to group interaction effect was observed (*P* = 0.03).

### 3.3. Effect of ALA Supplementation on ox-LDL and APO A1

At the baseline of the study, ox-LDL and APO A1 levels had no significant differences between the two groups. After supplementation with ALA, a significant reduction in ox-LDL (*P* < 0.001) and significant time to group interaction effect were observed (*P* < 0.001). There were no significant changes after intervention in APO A1 in both groups.

### 3.4. Effect of ALA Supplementation on Total Lp-PLA2 Mass and Its Distribution

**Figure 2:**
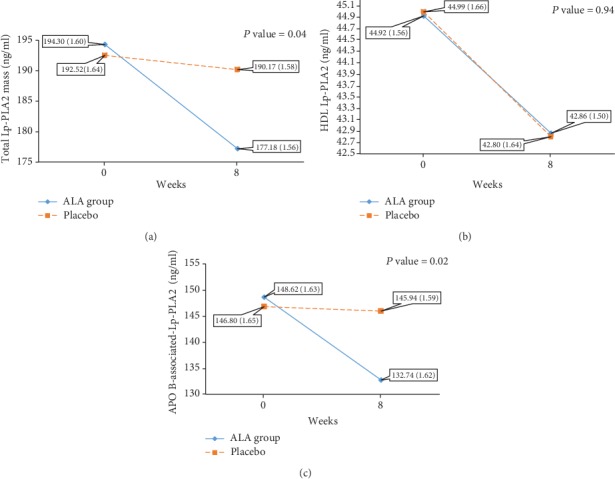
The changes in total Lp-PLA2 mass (a), HDL-Lp-PLA2 (b), and apoB-associated Lp-PLA2 (c) at the baseline and after 8 weeks of intervention. *P* value < 0.05 indicates a significant difference between groups based on the interaction effect shown by the two-way repeated measured ANOVA.

The changes in total Lp-PLA2 mass, HDL-Lp-PLA2, apoB-associated Lp-PLA2, and percent of HDL-Lp-PLA2 across the time are presented in Figure 2. At the baseline, the two groups were statistically similar in terms of these variables. ALA supplementation reduced total Lp-PLA2 mass (*P* = 0.001) mainly by decreasing the apoB-associated Lp-PLA2 (*P* = 0.001) not HDL-Lp-PLA2 (*P* = 0.25). The time to group interaction effect was significant with regard to total Lp-PLA2 mass (*P* = 0.019) and apoB-associated Lp-PLA2 (*P* = 0.01). HDL-Lp-PLA2 had a nonsignificant reduction in both groups; also, time to group interaction effect was not significant.

**Figure 3:**
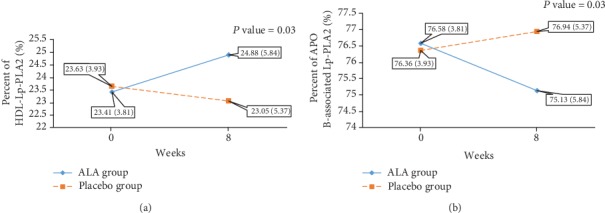
The changes in the percent of HDL-Lp-PLA2 (a) and the percent of apoB-associated Lp-PLA2 (b) at the baseline and after 8 weeks of intervention. Percent of HDL-Lp-PLA2: (HDL‐Lp‐PLA2/Lp‐PLA2 mass)∗100; percent of apoB-associated Lp-PLA2: (apoB‐associated Lp‐PLA2/Lp‐PLA2 mass)∗100. *P* value < 0.05 indicates a significant difference between groups based on the interaction effect shown by the two-way repeated measured ANOVA.

As shown in Figure 3, however, the percent of HDL-Lp-PLA2 increased nonsignificantly in the ALA group and the time to group interaction effect was significant (*P* = 0.03). It should be noted that, although the increase in the percent of HDL-Lp-PLA2 in the ALA group was not statistically significant, it was very close to the significant level given the *P* value (*P* = 0.051). Therefore, in the ALA group, the relative distribution of Lp-PLA2 improved by increasing the percent of HDL-Lp-PLA2 and a decrease in the percent of apoB-associated Lp-PLA2 compared to the placebo group.

### 3.5. Correlation Analysis

**Figure 4:**
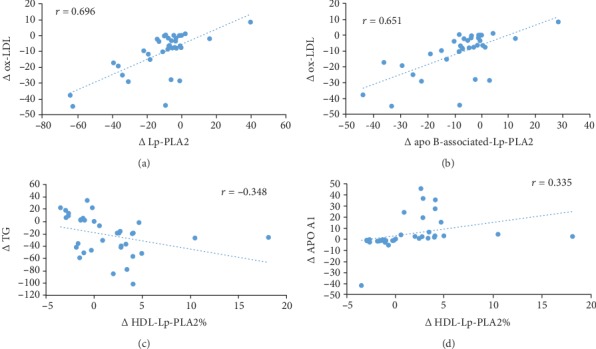
Correlation between changes of ox- LDL and total Lp-PLA2 mass (a), changes of ox-LDL and apoB-associated Lp-PLA2 mass (b), changes of TG and percent of HDL-Lp-PLA2 (c), and changes of APO A1 and percent of HDL-Lp-PLA2 (d) after supplementation with ALA.

To assess the correlation among outcomes, the Pearson correlation test was performed. At the baseline of the study, we found a positive correlation between total Lp-PLA2 mass with TC (*r* = 0.355, *P* = 0.003), LDL (*r* = 0.279, *P* = 0.019), and ox-LDL (*r* = 0.762, *P* < 0.001). In addition, apoB-associated Lp-PLA2 was positively correlated with TC (*r* = 0.361, *P* = 0.002), LDL (*r* = 0.292, *P* = 0.014), and ox-LDL (*r* = 0.756, *P* < 0.001) at the baseline of the study. As shown in Figure 4, there was a positive correlation among the changes of ox-LDL with changes of total Lp-PLA2 mass (*r* = 0.696, *P* < 0.001) and apoB-associated Lp-PLA2 (*r* = 0.651, *P* < 0.001) after supplementation with ALA. Furthermore, in the ALA group, the changes of percent of HDL-Lp-PLA2 were positively correlated with changes of APO A1 (*r* = 0.335, *P* = 0.049) but were negatively correlated with changes of TG (*r* = −0.348, *P* = 0.04).

### 3.6. Safety

According to a review article, supplementation with 600-2100 mg/day ALA has not associated with serious adverse health effects [32]. However, in some studies, side effects such as itching, urticaria, fever, and epigastria soreness (heart burning) have been reported in subjects taking the dosage of 1200 mg/day [46]. In our study, after supplementing with 1200 mg/day ALA for eight weeks, the participants had no complaints of side effects except one person who complained of heart burning after five weeks of supplementation. This problem disappeared after discontinuing the ALA supplement taking.

## 4. Discussion

The results of this double-blind randomized, parallel, placebo-controlled clinical trial revealed that supplementation with 1200 mg ALA for 8 weeks led to a significant reduction in total Lp-PLA2 mass, ox-LDL, and TG level and improved the distribution of Lp-PLA2 between HDL and apoB lipoproteins. Nevertheless, there was no significant effect on glycemic indices (such as FBS, insulin, and HOMA-IR), TC, HDL, LDL, and Apo A1 in type 2 diabetic patients.

Since at the baseline of the study, there were no substantial differences in medication distribution between the groups, and making any changes in medications three months before and during the study was one of the exclusion criteria, and in addition, there were no changes in the dietary intake and physical activity levels of participants during the study; therefore, our results can be attributed to ALA.

To our knowledge, it is the first clinical trial that examines the effect of ALA on Lp-PLA2 mass and its distribution among lipoproteins in type 2 diabetes patients.

ALA is synthesized de novo from medium-chain fatty acid, called octanoic acid, in small quantity in the body. ALA is found in food sources, such as red meat, the liver, the heart, and the kidney, and to a lesser degree, in spinach, broccoli, tomatoes, Brussel sprouts, potatoes, garden peas, and rice bran. There is evidence that food intake does not meet the appreciable amount of ALA; rather, oral dietary supplements are considered a primary source of ALA. The amount of ALA available in dietary supplements is about up to 1000 times more than the amount that could be obtained from diet. It has been shown that bioavailability of ALA from oral supplements is about 20-40%, while the efficiency of ALA absorption from the food source due to competition with other nutrients for carrier proteins is less. Therefore, it is recommended that ALA be taken 30 min before or 2 hours after a meal [27, 47].

Lp-PLA2, formerly named platelet-activating factor acetylhydrolase (PAF-AH), is a novel specific biomarker for vascular inflammation and useful tool for diagnosing and cardiovascular disease risk assessment in diabetic and general population. This enzyme may play a biological role in the development and progression of atherosclerosis [17, 48]. Lp-PLA2 catalyzes the hydrolysis of ox-LDL in the arterial wall and produces two inflammatory mediators, lysophosphatidylcholine and oxidized fatty acids [10, 19, 49]. Therefore, it appears that Lp-PLA2 may mediate the atherogenic effect of ox-LDL. According to the in vitro studies, inhibition of Lp-PLA2 diminished the arterial lesions evoked by ox-LDL [49, 50]. However, there is a controversy in the causal role of Lp-PLA2 in CHD. Some studies have shown that Lp-PLA2 was associated with CHD independent of conventional risk factors [13, 51, 52]. While in some GWAS, a variant in the PLA2G7 gene (V279F) that encodes the Lp-PLA2 with lower activity was not associated with cardiovascular risk markers, coronary atheroma, or CHD [11, 12].

In this study, 1200 mg ALA after eight weeks significantly reduced total Lp-PLA2 mass and ox-LDL levels compared with the placebo. Furthermore, we found a positive correlation between the reduction in ox-LDL level and total Lp-PLA2 mass in the ALA group. Such a positive correlation has also been observed in other clinical studies [53–55].

Ox-LDL is involved in the incidence and progression of atherosclerosis and diabetic complications. It has been reported that in diabetic patients, ox-LDL levels rise as the disease progresses, regardless of serum LDL levels [56]. ALA is a potent radical scavenger and protects against ROS-induced lipid oxidative damage [24]. In in vitro studies, ALA prevented the oxidation of LDL induced via oxidizing metals, such as Cu^2+^, by metal chelating activity and sparing vitamin E as the main antioxidant carried on LDL [36, 57]. In consistent with our study, supplementation with 600 mg ALA intravenously once daily for two weeks significantly decreased the plasma ox-LDL level in obese-impaired glucose tolerance patients [37].

The underlying mechanisms of the effects of ALA on Lp-PLA2 mass are unclear. It has been shown that ox-LDL is a potent stimulator of Lp-PLA2 expression by activating the P38 mitogen activating protein kinase (MAPK) pathway [49]. Therefore, the beneficial effects of ALA supplementation against Lp-PLA2 that are observed in our study might result from its reductive effects on ox-LDL serum levels. On the other hand, according to some in vitro studies, ALA attenuates P38 MAPK activation [58–60]. Then, we can consider the possibility that ALA indirectly downregulates Lp-PLA2 gene expression by suppressing the P38 MAPK activation. Furthermore, in an in vitro study, ALA suppressed Lp-PLA2 gene expression in THP-1 derived macrophages after stimulating these cells by H_2_O_2_ in order to induce the Lp-PLA2 gene expression [38].

The vast majority of plasma Lp-PLA2 is bound to apoB-containing lipoproteins, including LDL, VLDL, and IDL, and a smaller amount is associated with HDL; hence, apoB-associated Lp-PLA2 is the primary determinant of the total Lp-PLA2 level. It has been illustrated that HDL-Lp-PLA2 has anti-inflammatory and antiatherogenic properties, whereas apoB-associated Lp-PLA2 has proinflammatory activity [6]. A three-year follow-up prospective study in patients with stable CAD reported that higher HDL-Lp-PLA2 levels and a lower ratio of Lp-PLA2 mass to HDL-Lp-PLA2 are related to a lower risk for cardiac mortality, independent of other conventional cardiovascular risk factors [61]. According to these considerations, it has been suggested that the relative distribution of Lp-PLA2 may be a better cardiovascular risk marker rather than the total Lp-PLA2 level [23].

Darapladib, a selective Lp-PLA2 inhibitor, in both clinical trials and animal studies reduced the necrotic core volume and arterial lesions but did not reduce the rate of cardiovascular death, myocardial infarction, or stroke in patients with stable CHD [62–64]. It should be noted that all studies on darapladib have been focus on total Lp-PLA2 activity and there is no information on its effect on HDL-Lp-PLA2 and Lp-PLA2 distribution [6]. Since in an in vitro study inhibition of HDL-Lp-PLA2 by darapladib diminished the antioxidative and vasoprotective properties of HDL [65], it is likely that failure to reduce in CVD events be due to inhibition of HDL-Lp-PLA2 by darapladib and its effect on the Lp-PLA2 distribution.

In T2D patients, the impaired Lp-PLA2 distribution, which is characterized by lower HDL-Lp-PLA2 and higher apoB-associated Lp-PLA2, has been reported [17]. Passaro et al. showed that the incidence of type 2 diabetes might coincide with a shift of Lp-PLA2 towards the atherogenic lipoproteins particles [21].

As far as we know, this is the first study to investigate the effect of an oral nutritional supplement intervention on Lp-PLA2 distribution among lipoproteins. In our study, the decrease in total Lp-PLA2 after supplementation with ALA was associated with a significant decline in apoB-associated Lp-PLA2 and a nonsignificant reduction in HDL-Lp-PLA2, so resulting in an increase in the percent of HDL-Lp-PLA2 and a decrease in the percent of apoB-associated Lp-PLA2. These findings show that ALA improved the distribution of Lp-PLA2 among lipoproteins. In addition, we observed that in the ALA group, changes in the percent of HDL-Lp-PLA2 had a positive correlation with changes of APO A1 and a negative correlation with changes of TG.

It is unclear how ALA significantly increased the percent of HDL-Lp-PLA2 and improved the Lp-PLA2 distribution. Considering the reduction in the TG level and the negative correlation between TG changes and the percent of HDL-Lp-PLA2 after supplementation with ALA, one possible explanation is that since ALA may enhance the lipolysis of triglyceride-rich apoB-containing lipoproteins by activating the lipoprotein lipase (LPL) [37], probably, the Lp-PLA2s that bound to these lipolyzed lipoproteins have transferred to the HDL lipoproteins. Apparently, fibrates improve Lp-PLA2 distribution through such a mechanism [66].

APO A1 is the main structural protein of HDL and is a mediator in reverse cholesterol transport. In experimental and clinical studies, the upregulation of APO A1 was associated with a reduction in atherosclerosis [67]. APO AI overexpression resulted in a substantial increase in HDL-Lp-PLA2 in an animal model study [68]; in addition, the positive correlation between APO A1 and HDL-Lp-PLA2 has been reported [6, 69]. On the other hand, ALA may increase APO A1 synthesis in the liver [37]. So based on these evidences, we assumed that ALA possibly increase HDL-Lp-PLA2 and improve Lp-PLA2 distribution via APO A1 increase. However, in our study, the increase in APO A1 was not significant; this increase was positively correlated with the increase in the percent of HDL-Lp-PLA2.

According to in vitro studies, ALA stimulates translocation of glucose transporter 4 (GLUT4) to the membrane of fat and skeletal muscle cells, raises the activity of component of insulin signaling, and induces the expression of PPAR-1*α* and PPAR-1*γ*. Therefore, it may improve glucose hemostasis in diabetic patients [70, 71].

In our study, ALA supplementation could not improve the serum levels of glucose, insulin, and HOMA-IR. In accordance with our findings, de Oliviera et al. reported that treatment with 600 mg ALA for 20 weeks had no significant effect on serum levels of glucose, insulin, and HOMA-IR [72]. Likewise, Mendoza et al. found no significant reduction in serum glucose after supplementation with 600 mg ALA for six months [73]. In contrast, in one clinical study, after eight weeks of supplementation with 300 mg ALA in type 2 diabetic patients, an improvement in serum levels of glucose and HOMA-IR was reported [74]. Furthermore, supplementation with a food supplement containing 600 mg ALA after three months has shown a significant reduction in the serum level of glucose in patients with type 2 diabetes [31]. A possible explanation for our results is that all of our study population, similar to Oliveria's study, were well-controlled diabetic patients because one of our inclusion criteria was HbA1C ≤ 7% [72].

Little is known about the lipid-lowering effect of ALA in diabetic patients, and there are conflicting reports in this regard. In our study, after supplementation with 1200 mg ALA for eight weeks, we have found a nonsignificant effect on serum levels of TC, LDL, and HDL but a significant reduction in the TG level. Zhang et al. reported a significant reduction in serum levels of TC, LDL, and TG and a significant increase in HDL levels after intravenous administration of 600 mg ALA once daily for two weeks in obese-IGT patients [37]. In a study by Mendoza et al., after treatment with 600 mg ALA for six months in type 2 diabetic patients, a significant reduction in TC and an increase in HDL levels were reported; however, any significant change in TG levels was not observed [73]. In another study, supplementation with 600 mg ALA for 20 weeks in diabetic patients led to a nonsignificant effect on serum levels of TC, LDL, HDL, and TG [72].

The potential mechanism of the ALA effect on the lipid profile is not well understood. ALA may lower TC and LDL by [1] downregulating the squalene monooxygenase gene [2], increasing the LPL [3], synthesizing the LDL receptors in the liver which causes enhancing cholesterol uptake and increasing APO A1 synthesize [4], and inhibiting the oxidation of LDL to ox-LDL which improves the hepatic LDL receptor recognition and plasma clearance [32, 37]. In addition, ALA may improve TG levels via downregulating glycerol-3-phosphate acyltransferase, a rate-limiting enzyme of TG biosynthesize; enhancing lipolysis of TG-rich lipoproteins by activating LPL; and inhibiting the acetyl-CoA carboxylase activity [37, 75]. A meta-analysis study indicated that ALA supplementation resulted in a significant reduction in the serum level of TC, LDL, and TG but not on increasing the HDL level. According to this study, supplementation dosage, treatment duration, and baseline participants' BMI and lipid levels are determining factors that influence the lipid-lowering potential of ALA. More effects have been observed in higher ALA dosage (600 mg), treatment duration more than 12 weeks, and baseline BMI > 30 [75]. In our study, 82.9% of participants are taking statins and had normal serum levels of TC and LDL, so it appears they had less room for improvement. On the other hand, the participants mean baseline TG levels were higher than normal levels, and none of the participants were taking TG-lowering drugs such as fibrates. These can be a possible explanation for our results.

### 4.1. Limitations

One of the study limitations is that due to financial and time constraints, we could not investigate whether observed ALA positive effects are associated with the prevention of cardiovascular endpoints and atherosclerotic lesions determined either by ultrasonography or by angiographic techniques. Further, since the laboratory methods used to measure the blood ALA levels are complex and costly, we had not been able to evaluate patient compliance accurately. However, we compensated this limitation to some extent through frequent and regular telephone calls and counting the remaining capsules, so we observed a 98% compliance rate in these patients. Another limitation of our study was using 24 h recall instead of food record to assess the dietary intake due to the limited literacy skill of some participants to measure or judge the food portion size and probability of noncompliance with the food record submission. Also, unfortunately, because of the participants' commuting difficulties, it was not possible to have more visits to obtain 24 h recall during the study.

## 5. Conclusion

In this double-blind, randomized, parallel, placebo-controlled clinical trial, supplementation with 1200 mg ALA after eight weeks resulted in a substantial reduction in ox-LDL and Lp-PLA2 mass and improvement in Lp-PLA2 distribution between HDL and apoB-containing lipoproteins in type 2 diabetic patients. These observed desired effects may be associated with the reduction in the risk of CVD in these patients. Further clinical trial studies should be conducted to evaluate the cardiovascular endpoints and cardioprotective effects of this supplement in the long term.

## Figures and Tables

**Table 1:** Baseline characteristics of participants.

Variable	ALA group (*n* = 35)	Placebo group (*n* = 35)	*P* value
Age (year)^a^	52.66 (4.81)	53.34 (4.45)	0.53
Female/male^b^	20 (57.1)/15 (42.9)	19 (54.3)/16 (45.7)	0.81
Menopause ^b^	13 (37.1)	14 (40)	0.81
Duration of diabetes (year)^a^	3.65 (1.67)	3.57 (1.83)	0.83
HbA1C^a^	6.6 (0.38)	6.7 (0.21)	0.17
BMI^a^	25.4 (1.25)	24.93 (1.26)	0.11
Medication^b^			
Metformin	32 (91.4)	31 (88.6)	0.69
Sulfonylurea	8 (22.9)	10 (28.6)	0.58
ARB	28 (80)	27 (77.1)	0.77
ACEI	4 (11.4)	5(14.3)	0.72
Statins	29 (82.9)	30 (85.7)	0.74
Aspirin	29 (82.9)	27 (77.1)	0.55
Physical activity level^b^			
Low	20 (57.1)	21 (60)	0.80
Moderate	15 (42.9)	14 (40)	

^a^Presented as mean (SD), *P* values are resulted from independent sample t-test. ^b^Presented as number (%), *P* values are resulted from a chi-square test. Low physical activity: score less than 600 MET-minutes/week and moderate physical activity: score between 600 and 3000 MET-minutes/week.

**Table 2:** Dietary intake of participants at the baseline and after 8 weeks of intervention.

Variables		ALA group	Placebo group	*P* value^∗^	*P* value^∗∗^
Energy (kcal/day)	Before	2227.57 (363.28)	2269.74 (356.95)	0.62	0.62
	After	2176.22 (282.93)	2240.28 (345.06)		
Carbohydrate (g/day)	Before	302.61 (52.31)	307.73 (49.54)	0.67	0.89
	After	303.17 (50.76)	309.06 (47.16)		
Carbohydrate (%)	Before	54.28 (2.46)	54.24 (2.40)	0.94	0.86
	After	55.60 (4.63)	55.37 (4.80)		
Protein (g/day)	Before	61.20 (9.28)	60.31 (10.48)	0.70	0.33
	After	60.02 (7.49)	60.66 (9.87)		
Protein (%)	Before	11.03 (0.74)	10.64 (0.94)	0.06	0.43
	After	11.04 (0.41)	10.82 (0.54)		
Fat (g/day)	Before	85.80 (14.91)	88.61 (15.99)	0.45	0.72
	After	80.38 (14.08)	84.59 (19.81)		
Fat (%)	Before	34.67 (2.37)	35.10 (2.36)	0.45	0.98
	After	33.34 (4.61)	33.79 (4.90)		
Vitamin A (*μ*g/day)	Before	646.30 (132.88)	618.13 (92.49)	0.30	0.66
	After	647.51 (126.12)	622.71 (100.52)		
Vitamin E (mg/day)	Before	18.63 (4.12)	19.23 (4.20)	0.54	0.34
	After	18.20 (3.82)	18.15 (4.51)		
Vitamin C (mg/day)	Before	79.20 (10.50)	83.05 (13.06)	0.17	0.43
	After	81.36 (10.66)	86.42 (15.18)		
Riboflavin (mg/day)	Before	1.60 (0.43)	1.63 (0.49)	0.74	0.86
	After	1.59 (0.40)	1.62 (0.52)		
Zinc (mg/day)	Before	9.68 (1.93)	9.95 (2.20)	0.59	0.84
	After	9.94 (1.94)	10.28 (2.23)		
Selenium (*μ*g/day)	Before	67.06 (11.59)	64.09 (15.16)	0.36	0.75
	After	67.83 (11.91)	65.17 (15.80)		
Iron (mg/day)	Before	8.89 (2.66)	9.67 (3.50)	0.29	0.75
	After	9.14 (2.25)	10.06 (2.65)		

The results are described as mean (SD). ^∗^according to independent *t*-test at baseline; ^∗∗^time to group interaction according to two-way repeated measure ANOVA.

**Table 3:** BMI and biochemical parameters of participants at the baseline and after 8 weeks of intervention.

Variables		Before	After	*P* value^∗^	*P* value^∗∗^
BMI (kg/m^2^)	ALA group	25.41 (1.25)	25.32 (1.27)	0.13	0.16
	Placebo group	24.93 (1.26)	2ox4.99 (1.15)	0.52	
APO A1 (mg/dl)	ALA group	123.56 (18.99)	128.34 (19.42)	0.06	0.09
	Placebo group	126.17 (22.13)	123.00 (22.62)	0.42	
Ox-LDL (ng/l)	ALA group	42.82 (1.44)	32.14 (1.46)	0.00	0.00
	Placebo group	35.89 (1.61)	34.43 (1.71)	0.19	
TC (mg/dl)	ALA group	159.91 (24.22)	151.74 (21.92)	0.15	0.30
	Placebo group	153.63 (21.00)	151.69 (17.80)	0.43	
HDL (mg/dl)	ALA group	47.43 (5.87)	45.74 (7.64)	0.25	0.98
	Placebo group	45.37 (4.88)	43.71 (6.87)	0.26	
LDL (mg/dl)	ALA group	78.18 (22.74)	76.01 (23.37)	0.66	0.72
	Placebo group	71.94 (18.70)	71.66 (13.38)	0.89	
TG (mg/dl)	ALA group	171.26 (31.86)	149.03 (39.23)	0.00	0.03
	Placebo group	181.14 (50.42)	180.11 (52.18)	0.89	
FBS (mg/dl)	ALA group	134.03 (11.56)	127.74 (15.26)	0.07	0.06
	Placebo group	131.51 (14.92)	133.51 (11.71)	0.48	
Insulin (mU/l)	ALA group	10.83 (1.53)	10.74 (1.55)	0.54	0.38
	Placebo group	10.94 (1.63)	11.12 (1.14)	0.51	
HOMA-IR	ALA group	3.58 (0.58)	3.38 (0.62)	0.07	0.058
	Placebo group	3.55 (0.66)	3.67 (0.53)	0.34	

The results are described as mean (SD). BMI: body mass index; Apo A1: apolipoprotein A1; ox-LDL: oxidized low-density lipoprotein; TC: total cholesterol; HDL: high-density lipoprotein cholesterol; LDL: low-density lipoprotein cholesterol; TG: triglyceride; FBS: fasting blood sugar; HOMA-IR: homeostasis model of assessment index. ^∗^according to paired t-test; ^∗∗^time to group interaction according to two-way repeated measure ANOVA.

## Data Availability

The data (database in Excel) used to support the findings of this study are available from the corresponding or first author upon request. (saeedhmdphd@hotmail.com, n-baziar@razi.tums.ac.ir)
